# An Integrated Framework for Automated Image Segmentation and Personalized Wall Stress Estimation of Abdominal Aortic Aneurysms

**DOI:** 10.21203/rs.3.rs-6630234/v1

**Published:** 2025-06-12

**Authors:** Merjulah Roby, Juan C. Restrepo, Deepak K. Shan, Satish C. Muluk, Mark K. Eskandari, Vikram S. Kashyap, Ender A. Finol

**Affiliations:** 1 Department of Mechanical, Aerospace, and Industrial Engineering, The University of Texas at San Antonio, San Antonio TX, 78249, USA; 2 Department of Thoracic and Cardiovascular Surgery, Allegheny Health Network, Allegheny General Hospital, Pittsburgh PA, 15212, USA; 3 Northwestern University, Feinberg School of Medicine, Chicago IL, 60611, USA; 4 Cardiovascular Health, Corewell Health, Grand Rapids MI, 49503, USA

## Abstract

Abdominal Aortic Aneurysm (AAA) remains a significant public health challenge, with an 82.1% increase in related fatalities from 1990 to 2019. In the United States alone, AAA complications resulted in an estimated 13,640 deaths between 2018 and 2021. In clinical practice, computed tomography angiography (CTA) is the primary imaging modality for monitoring and pre-surgical planning of AAA patients. CTA provides high-resolution vascular imaging, enabling detailed assessments of aneurysm morphology and informing critical clinical decisions. However, manual segmentation of CTA images is labor intensive and time consuming, underscoring the need for automated segmentation algorithms, particularly when feature extraction from clinical images can inform treatment decisions. We propose a framework to automatically segment the outer wall of the abdominal aorta from CTA images and estimate AAA wall stress. Our approach employs a patch-based dilated modified U-Net model to accurately delineate the outer wall boundary of AAAs and and Nonlinear Elastic Membrane Analysis (NEMA) to estimate their wall stress. We further integrate Non-Uniform Rational B-Splines (NURBS) to refine the segmentation. During prediction, our deep learning architecture requires 17 ° 0.02 milliseconds per frame to generate the final segmented output. The latter is used to provide critical insight into the biomechanical state of stress of an AAA. This modeling strategy merges advanced deep learning architecture, the precision of NURBS, and the advantages of NEMA to deliver a robust, accurate, and efficient method for computational analysis of AAAs.

## Introduction

AAA is a serious condition in which the abdominal segment of the aorta dilates abnormally. If it ruptures, the mortality rate can be up to 80%. The probability of developing an AAA increases with age, smoking, high blood pressure, plaque formation (atherosclerosis), and male sex^1,2^. AAAs often do not present symptoms in their early stages, making detection difficult without screening. However, as the aneurysm expands, it can cause severe abdominal or back pain. In clinical practice, vascular surgeons or interventional radiologists use the maximum diameter of the aneurysm to estimate its future risk of rupture^3^. Current guidelines recommend surgery for men if the aneurysm is at least 5.5 cm in diameter and for women if it is in the range of 5.0 to 5.4 cm in diameter^2^.

AAAs are typically diagnosed using various imaging modalities, including ultrasound, computed tomography angiography (CTA), and magnetic resonance imaging (MRI). CTA is essential in the diagnosis, treatment, and surveillance planning of AAAs, as it produces highly comprehensive three-dimensional images of the aorta and surrounding tissues. These images are necessary to accurately assess the dimensions and morphology of the aneurysm, which are used to decide the need for repair. CTA image segmentation, which involves isolating specific regions of interest (ROIs), can be carried out using semi-automated or fully automatic procedures.

Several advances have been made in the development of clinical image segmentation methods to identify aortic aneurysms. Kurugol *et al.*^4^ used unenhanced CTA images to quantify the shape of an AAA by combining anatomical localization, circular Hough transform, and 3D level set segmentation. Shang *et al.*^5^ utilized a semi-automated method to monitor the AAA wall by recognizing isointensity contours in CTA images. De Bruijne *et al.*^6^ proposed manual delineation followed by automatic segmentation of CTA images using a statistical shape model. Wang *et al.*^7^ improved the geodesic active contour approach to segment AAAs using MRI data. Herment *et al.*^8^ proposed a 2D deformable surface model to segment the thoracic aorta in MR images. Xie *et al.*^9^ created an algorithm that uses anatomical localization and cylinder tracking to segment the thoracic aorta in unenhanced CTA images.

Before deep learning techniques were developed, semiautomated approaches for image segmentation were implemented. Although these methods yielded reasonable results, they often required extensive fine-tuning to achieve optimal precision. Given the limitations of the aforementioned approaches, the need for automated segmentation tools for medical image analysis is warranted. Chandrashekar *et al.*^10^ used deep learning to automatically segment aortic aneurysms in CTA images, achieving DSC (Dice Similarity Coefficient) scores of 0.887 ± 0.005 at low resolution and 0.932 ± 0.007 at high resolution. Lyu *et al.*^11^ compared the performance of two advanced models, ARU-Net and CACU-Net, and found that they achieved DSC scores of 0.916 ± 0.028 and 0.916 ± 0.029, respectively. Kim *et al.*^12^ used a CNN-based segmentation technique (Convolutional Neural Network) (developed by Zhang *et al.*^13^) to identify the lumen and outer wall from 3D CTA scans, resulting in a DSC score of 0.866 ± 0.044. These techniques have significantly improved AAA segmentation and while future tests on larger and more complex cases will help demonstrate their effectiveness in clinical practice, the models need to be fine-tuned to improve their accuracy and reliability.

As an alternative risk assessment surrogate beyond the maximum diameter, peak wall stress can be estimated using 3D geometries generated after image segmentation using Finite Element Analysis (FEA)^14^. However, predefined constitutive material properties are required as input to traditional forward FEA simulations. These material properties are typically derived from uniaxial and biaxial tensile tests of ex vivo tissue samples, with material models based on assumptions about different biomechanical behaviors, such as isotropic or anisotropic characteristics in elastic or hyperelastic material frameworks^15^. Despite a large sample base, material behavior can vary in vivo and regionally on a subject-specific basis, potentially leading to inaccurate wall stress estimates. For example, the tensile strength of ruptured AAA tissue has been found to be lower than that of tissue obtained from electively repaired AAAs (54 N/cm^2^ vs. 84 N/cm^2^)^16^. For patients in surveillance programs, it is not yet possible to characterize AAA tissue non-invasively. Conversely, alternate solutions have been proposed to overcome the limitation of the predefined material properties required for FEA. Abdominal / pelvic CTA images represent a deformed configuration of the abdominal aorta, commonly under mean arterial pressure in which static determinancy is maintained. Inverse methods^17–22^ have been developed to calculate wall stress based on this deformed configuration with known applied loads via membrane equilibrium, which eliminates the need to specify material properties for the AAA wall^23,24^.

The primary objective of this work is to develop a framework using a novel image segmentation strategy that meets the demand for automatic segmentation and precision integrated with a wall stress estimation procedure. Combined, these methods enable the creation of AAA models that are used to estimate AAA wall stress using a non-linear elastic membrane model without user intervention, overcoming the need for pre-defined material properties. A secondary objective is the development of a user interface for the optional manual adjustment of the segmentation results using NURBS. This tool enhances the automatic segmentation model, achieving ground-truth-like accuracy and ensuring the reliability required for diagnostic and therapeutic processes, especially in complex anatomical cases.

## Methods

### Study Subjects

We retrospectively collected 147 CTA exams from 76 symptomatic and 71 asymptomatic patients at Allegheny Health Network and Northwestern Memorial Hospital, after approval from the Institutional Review Boards (IRBs) of both medical centers. All methods were performed in accordance with the relevant guidelines and regulations of the respective IRBs. Consequently, informed consent from the patients was not required, as this study consisted of a retrospective review of existing deidentified clinical data. The data set includes 9,037 images, which were semi-automatically segmented with the in-house tool AAAVasc, validated previously^25^. The data set represents ground-truth data for training and testing the accuracy of the framework’s automated segmentation algorithm. In addition, we used 35 CTA datasets from 12 AAA participants in a surveillance program to use the framework for the prediction of wall stress.

### Segmentation of the AAA Outer Wall

We based the 2D U-Net architecture on Ronneberger’s *et al.*^26^ original U-Net model, optimizing it specifically for CTA image segmentation. Its encoder-decoder construction resembles a “U,” which helps in the extraction of detailed features and the generation of high-quality output. In this architecture, dilation rates are critical to how the network interprets images in its convolutional layers. These rates describe the spacing between pixels in convolutional filters. A higher dilation rate indicates the filters are farther apart, allowing them to gather information from a larger area of the image. The effective receptive field of a dilated convolution is defined by Eq. (1),

(1) $$RF=\left(k-1\right)\cdot d+1$$

where d represents the dilation rate and k the kernel size. In a conventional CNN layer, convolutions use a fixed d=1, which means that each kernel works on adjacent pixels. This limits the receptive field; to capture a larger context, deeper networks or pooling mechanisms are required. In contrast, dilation enables the network to detect long-range correlations while maintaining spatial resolution by increasing the receptive area without introducing new parameters. This is particularly useful in medical image segmentation, where exact identification of minute anatomical details is critical.

**Figure 1.**
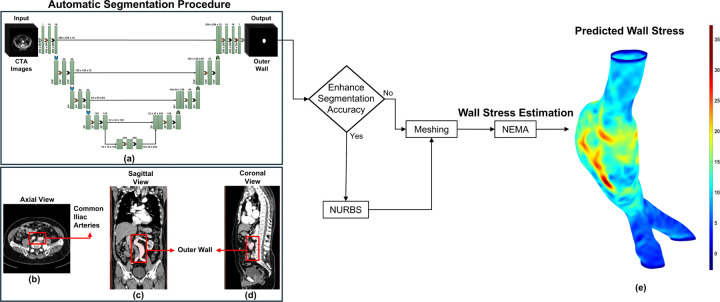
Framework for automated segmentation and personalized wall stress in AAAs: (a) Architecture of the patch-based dilated U-Net model; (b) Exemplary binary masks overlaid on an AAA image; (c) and (d) Reconstruction and visualization of the AAA in the coronal and sagittal planes; (e) First principal wall stress distribution in a mesh generated using the automated segmentation; stress values are in N/cm^2^.

In our U-Net design, we intentionally alter the dilation rates between layers. We begin with a dilation rate of one, meaning that the filters scrutinize each pixel closely. Subsequently, we increase the rates to 2 and 3, before returning to 1. By gradually changing the dilation rate, our model identifies both local and global data more effectively than typical CNN designs, resulting in higher segmentation accuracy. This method is beneficial for feature extraction, since it enables the network to detect and analyze complicated patterns and structures in the image. Figure 1 (a) illustrates the overall study design of the U-Net architecture for clinical image segmentation. In addition to architectural components, our methodology includes patch-based processing. This technique breaks large image stacks into smaller, more manageable patches that are processed individually. It offers quick analysis and segmentation of large datasets, allowing complete analysis while managing computing complexity. To obtain the best border delineation, the segmentation model is trained using a combination of the Dice loss function and binary cross-entropy (BCE) loss, which balances pixel-wise classification confidence (BCE loss) with area overlap accuracy (Dice loss). Furthermore, data augmentation procedures are used to expand the training dataset and improve the generalization of the model. Augmentation techniques such as horizontal and vertical flipping, rotation, intensity scaling, and zoom transformations were applied to make the model more adaptable and accurate when analyzing new unseen images. It also reduces overfitting by using several representations of the training data, improving the ability to handle additional unknown data during inference. Additional methodological details are provided elsewhere^27^.

### User-interactive NURBS-based Tool

We created a user-friendly tool using NURBS^28^, which was developed as an interactive Python application with a graphical user interface created using Tkinter. It enables users to input CTA images and their related segmentation masks, and manually fine-tune segmentation boundaries by modifying interactive points on the contours. This real-time user intervention can be critical for medical experts who need to make rapid changes so that the contours accurately represent their anatomical features of choice. Creating a NURBS curve using control points requires calculating a knot vector, which affects the manner the curve transitions between points. The curve is then evaluated at particular locations along its length using Eq. (2),

(2) $$c\left(t\right)=\frac{\sum_{i=0}^{n}N_{i,p}\left(t\right)w_{i}P_{i}}{\sum_{i=0}^{n}N_{i,p}\left(t\right)w_{i}}$$

where $c\left(t\right)$ is the point on the NURBS curve at parameter u; ${N_{i}}_{,p}\left(t\right)$ is the $i^{th}$ B-spline basis function of degree p; $w_{i}$ is the weight associated with the $i^{th}$ control point; $P_{i}$ is the $i^{th}$ control point; and t is the parameter, which varies from 0 to 1 along the length of the curve.

The NURBS tool allows users to manually fine-tune image segmentation by modifying contour points and adding new ones as necessary. It also offers a technique for improving segmentation masks using smooth curves, which yield more precise shapes. Users can proceed with NURBS-based changes or revert to automatic segmentation results. The technique enables users to make informed decisions about how automated forecasts or NURBS modifications may affect future treatment suggestions.

### Nonlinear Elastic Membrane Analysis (NEMA) for Wall Stress Estimation

Pressure vessels are statically determined even when they have thick walls^29^. Therefore, intramural stress depends only on the geometry and internal load, allowing AAA wall stress to be determined by solving for elastic determinancy^17^. This approach enables the estimation of wall stress without relying on predefined material properties. In this analysis, the equilibrium equations were solved for a deformed configuration of the AAA. Using linear shape functions to represent the spatial variation of the components of the equilibrium equation, i.e. Eq. (3), effectively yields a set of non-linear algebraic equations^21^ in $\sigma^{\alpha \beta }$. Consequently, the components of the Cauchy stress tensor can be evaluated at the centroid of each element^17,18,24,30^ and the aforementioned algebraic equations are solved using the inverse method,

(3) $$\frac{1}{\sqrt{g}}{\left(\sqrt{g}h\sigma^{\alpha \beta }g_{\alpha }\right)}_{,\beta }+pn=0$$

where g: determinant of the metric tensor; h: element thickness; $\sigma^{\alpha \beta }$: Cauchy stress components to be determined in the local contravariant basis (α,β=1,2); and p: intraluminal pressure.

NEMA takes as input a 3D triangular surface mesh representing the outermost layer of the wall in its deformed configuration—under mean arterial pressure—with a specified wall thickness. Intraluminal pressure was applied outwards to the internal faces of the mesh elements, while the distal and proximal ends were fixed. These 3D meshes were constructed using the AAAMesh custom script^31^ using CTA-specific slice spacing and pixel size. NEMA was previously validated by Thirugnanasambandam *et al.*^32^ using a benchtop flow loop with a deformable AAA silicone phantom of a patient-specific geometry. The phantom was imaged using MRI to acquire time-resolved images of the wall. From these images, a 3D geometry was generated at a peak systolic pressure of 140 mmHg, which was the internal load for NEMA. The results were compared to those of an FEA simulation using an isotropic linear elastic material on the zero-pressure geometry derived from the MRI data^24^.

In this work, we applied NEMA to predict the first principal wall stress for 105 surface meshes from 12 AAA patients under surveillance (derived from 35 CTA exams) in three groups: Ground Truth, Predicted, and Predicted + NURBS. Each mesh had a uniform wall thickness of 1.5 mm and was subjected to a mean arterial pressure of 93.3 mmHg with fixed proximal and distal ends. Figure 1 shows the integrated framework for automated segmentation and personalized wall stress estimation in AAAs. Figures 1 (b)–(d) show an exemplary result of the outer wall boundaries of an AAA. Figure 1 (b) displays the axial plane of an initial CTA image overlayed with the binary mask, while Figures 1 (c) and 1 (d) depict the coronal and sagittal planes, respectively. Figure 1 (e) shows the first principal wall stress distribution predicted using NEMA.

## Results

### Identification of the Outer Wall Boundary

**Figure 2.**
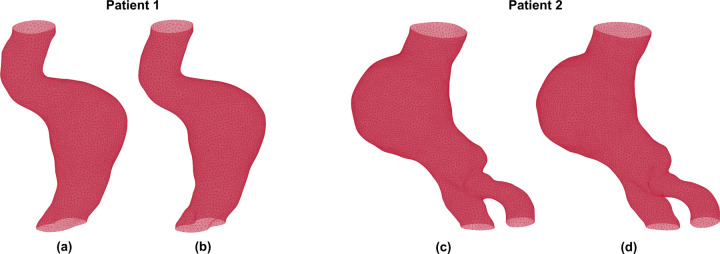
Volume meshes generated from the ground truth [(a) and (c)] and the predicted [(b) and (d)] image segmentations.

The boundary of the outer wall was detected accurately using the patch-based dilated U-Net model, which was trained on 9,037 contrast-enhanced CT images and tested on an additional 1,063 images. To assess segmentation accuracy, the output of the model, i.e. the predicted outer wall boundaries, was compared to the ground truth obtained from previously segmented images using AAAVasc. Figure 2 shows visual comparisons of volume meshes generated from ground truth and predicted segmentations for patients 1 and 2. Figures 2 (a) and 2 (c) show the ground truth volume meshes while Figures 2 (b) and 2 (d) show the predicted volume meshes, respectively. This comparison illustrates the qualitative differences and similarities that are evident by visual inspection of the 3D geometries resulting from the ground truth and predicted segmentations.

### Performance Metrics for Segmentation Assessment

We used eight metrics to evaluate the performance of the U-Net segmentation model computed using the test data set of 1,063 images to evaluate the efficiency of the model in the unseen data set. The model identified and delineated the AAA boundaries of interest for the outer wall segmentation, with an accuracy of 99.96%. A sensitivity score of 97.28% demonstrates the ability of the model to detect true positives accurately. The model’s precision of 96.69% demonstrates its ability to detect targeted structures accurately with few false positives. The Matthews Correlation Coefficient (MCC) of 0.9695 indicates a strong correlation between predicted and ground-truth segmentations. The DSC of 0.9695 demonstrates the model’s ability to produce consistent and accurate overlapping segmentations. The model’s specificity of 99.98% indicates that it eliminates unnecessary areas in the segmentation ROIs. An IOU score of 94.11% demonstrates its ability to accurately segment the outer wall and handle complex anatomical components. The mean 95% Hausdorff Distance (HD) is 1.84 mm, which is the maximum distance between the predicted segmentation and the ground truth. The 95% HD metric is commonly used in medical imaging to reduce outliers by reporting the 95th percentile of distances. It is the largest distance between a point in a set and the nearest point in another. Additional metrics and a comparison with other segmentation methods were reported in^27^.

### Maximum AAA Diameter

**Figure 3.**
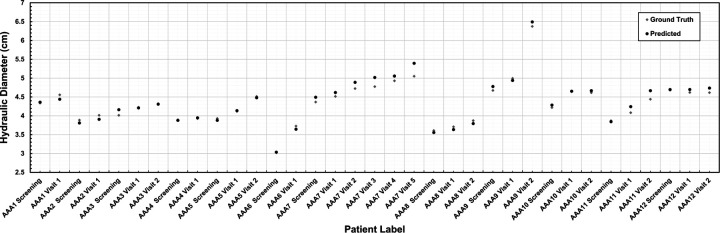
Comparative analysis of the maximum hydraulic diameter (in cm) measured using the outer wall boundary.

We evaluated the segmentation model’s accuracy using the maximum diameter to assess suitability for a clinical application. This was performed by comparing the maximum hydraulic diameter obtained using two different segmentation methods: expert medical segmentation (ground truth) and predictive algorithmic segmentation. Figure 3 illustrates this comparison for the 12 surveillance patients at each of the CTA imaging follow-ups (initial screening and one or more subsequent visits), which produced 35 measurements of maximum diameter. The average absolute difference between the ground truth and the predicted maximum diameter is 0.0424 cm.

### Metal Artifacts

**Figure 4.**
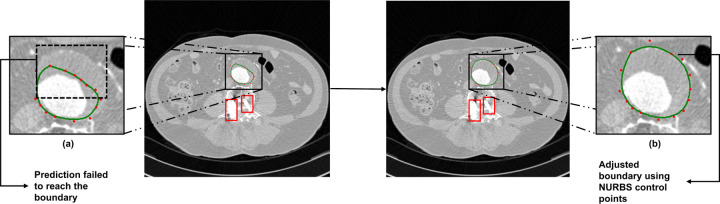
Segmentation of a complex AAA case with metal artifacts: (a) Prediction failure due to metal artifacts (red boxes) affecting automated segmentation and (b) Corrected segmentation using control points of the NURBS curve.

In a complex AAA case, follow-up CT images show the presence of a metal artifact, specifically a spinal screw, which caused the automated segmentation model to incorrectly identify the boundary of the outer wall. This follow-up CT examination includes 142 images, with the model failing to segment the outer wall correctly in six of the images. Figure 4 (a) shows the original segmentation output of the automated model, where metal artifacts (highlighted in red boxes) led to inaccuracies in segmentation. In contrast, Figure 4 (b) illustrates the improved boundary detection achieved by manually setting control points on the NURBS curve. This refinement is essential to improve the outcome of the segmentation of complex cases such as those with metal artifacts, ensuring that the outer wall boundary meets the interventional radiologist’s or vascular surgeon’s requirement and achieves a high level of precision.

### Wall Stress Estimation

**Figure 5.**
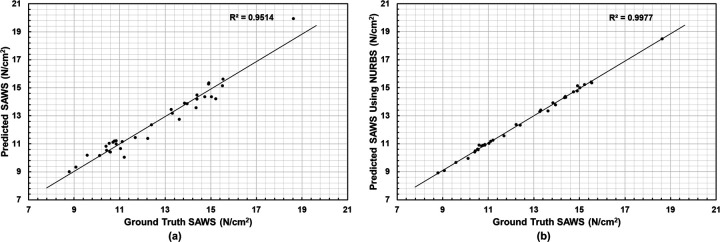
SAWS correlation relative to the Ground Truth calculated with Predicted (a) and Predicted + NURBS (b) meshes.

From the NEMA stress distributions, five biomechanical parameters were calculated, namely Peak Wall Stress, the 99th and 75th Percentile Wall Stress, Mean Wall Stress, and the Spatially Averaged Wall Stress (SAWS). The latter was calculated as the area-weighted average of wall stress using the surface areas of the mesh elements. We compared the predicted wall stress relative to the ground truth wall stress using the coefficient of determination $R^{2}$. Figure 5 (a) shows the correlation for SAWS obtained from the Predicted and Ground Truth groups, leading to a $R^{2}=0.9514$. For nearly half of the 35 AAA geometries, the Predicted SAWS (obtained from surface meshes generated from the U-Net patch-based model segmentations) underestimated the Ground Truth SAWS (obtained from surface meshes generated by the ground truth segmentations using AAAVasc). Similarly, Figure 5 (b) illustrates an excellent agreement between the Predicted SAWS using NURBS (obtained from surface meshes generated from the U-Net patch-based model segmentations corrected with the interactive NURBS tool) and the Ground Truth SAWS. This is evident from the high coefficient of determination $\left(R^{2}=0.9977\right)$ between these two groups.

**Figure 6.**
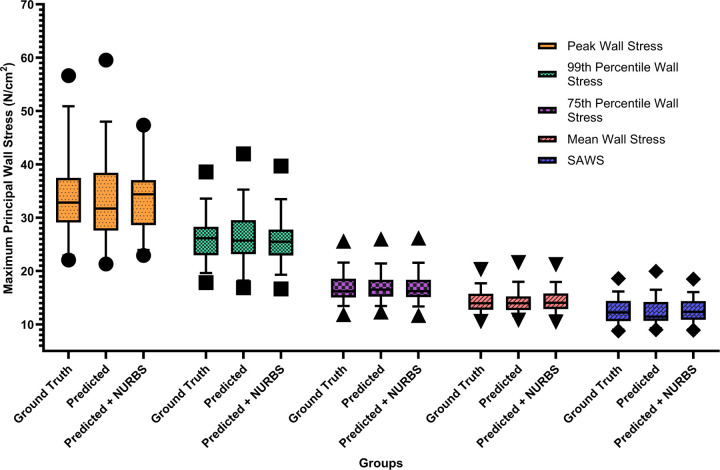
Box and whisker plots for the three mesh-based groups comparing five biomechanical parameters.

**Figure 7.**
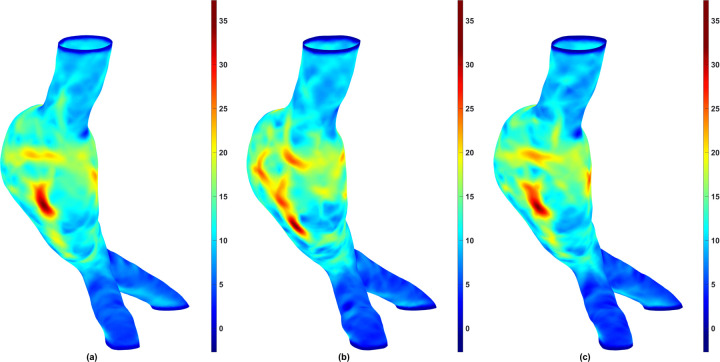
Maximum principal wall stress distribution for an exemplary AAA representative of the Ground Truth (a), Predicted (b), and Predicted + NURBS (c) groups. Stress values are in N/cm^2^.

To evaluate whether the differences in biomechanical parameters obtained for the three aforementioned groups were statistically significant, we performed a Friedman test (Friedman Statistic, FS) with a significance level of α = 0.05 using GraphPad Prism (GraphPad Software, Boston, MA). This approach enabled pairwise comparisons between the Predicted and Predicted + NURBS groups against the Ground Truth group. Figure 6 shows the box-and-whisker plot for each biomechanical parameter for the three groups. The Friedman test revealed that there is no statistically significant difference between the groups for any parameter: Maximum Wall Stress (FS = 0.7429, *p* = 0.6897), 99th Percentile Wall Stress (FS = 4.171, *p* = 0.1242), 75th Percentile Wall Stress (FS = 3.657, *p* = 0.1606), Mean Wall Stress (FS = 1.086, *p* = 0.5811), and SAWS (FS = 1.086, *p* = 0.5811). The relative similarity revealed by the Friedman test can be observed using a stress map on the surface meshes. Figure 7 shows color maps of the spatial distribution of the first principal wall stress for an exemplary AAA geometry using (a) the Ground Truth mesh; (b) the mesh generated directly from the predicted segmentation; and (c) the mesh generated after applying NURBS on the predicted segmentation.

## Discussion

In clinical practice, measuring the maximum diameter of AAA is frequently performed manually, which could lead to variability between observers. To address this shortcoming, we developed a deep learning model for automatically segmenting abdominal CTA images that identify the outer wall boundary of an AAA. An interactive NURBS-based tool improves the accuracy of the model, which is especially useful for addressing challenging cases that include metal artifacts in images. Performance metrics reveal that the model is highly accurate, suggesting its potential for use in a clinical setting as part of a risk assessment modeling pipeline. The output of the segmentation model was subsequently used to create in-silico models of 12 AAA patients under surveillance with multiple imaging follow-ups. These patient-specific models provide an evaluation of the biomechanical state of each AAA over time, which in turn can be used by the treating physician to make an informed decision about recommending AAA repair.

Compared to existing automatic segmentation approaches, the proposed patch segmentation method has several advantages. The traditional deep learning models use standard convolutions with a confined receptive field; our model’s dilated convolutions efficiently enhance the receptive field without increasing processing costs. This enables more precise segmentation of complex anatomical structures and improved feature extraction. Furthermore, unlike existing segmentation algorithms, which often process complete images at once, our patch-based methodology provides greater resolution feature extraction by breaking down images into smaller ones. The accuracy of the model, with a DSC of 96.95%, outperforms existing CNN-based techniques^27^.

**Figure 8.**
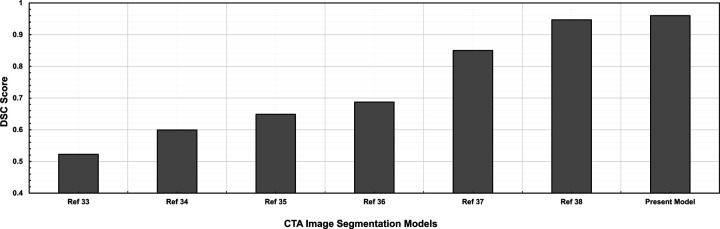
Comparative analysis of Dice Score Coefficient: existing models vs. present model.

This increased segmentation reliability reduces false positives and false negatives, making the findings more therapeutically relevant. The fully automated segmentation pipeline predicts an image in 17 ± 0.02 milliseconds, thereby eliminating the need for manual correction. This greatly reduces processing time compared to existing approaches that require manual adjustment or additional fine-tuning steps. Finally, our proposed model performs well in difficult cases, such as those with metal artifacts or tortuous aneurysms. The performance comparison of our model with existing methods^33–38^, based on the DSC, is shown in Figure 8. When necessary, the NURBS-based refinement tool enables accurate manual correction, further improving segmentation accuracy, while the patch-based technique provides flexibility in these difficult cases. Depending on the case’s complexity, the average NURBS processing time for one image ranges from 3 to 20 seconds.

Lareyre *et al.*^39^ developed an automated pipeline for AAA lumen segmentation employing feature-based techniques, including boundary propagation and active contour methods. Although their method performed well on a data set of 40 CTAs, it produced false positives and negatives, especially when identifying the aorta and renal arteries. Their pipeline is time efficient, while segmenting the lumen in less than 1 minute per patient. In contrast, the implementation of a NURBS-based tool for users to interactively modify segmentation boundaries results in anatomically correct representations of the aortic wall based on the user’s choice. The NURBS-enhanced model reduced the image processing time by 90% compared to the ground-truth method, namely the segmentation provided by AAAVasc, making it more appealing for real-time clinical applications where speed and accuracy are important.

The analysis of five biomechanical parameters for the three groups revealed quantifiable differences in wall stress estimates; however, these differences were not found to be statistically significant. We infer from this result that the automated segmentation model with or without NURBS corrections yielded in-silico AAA models that were statistically similar to the Ground Truth for wall stress estimation (for the surveillance AAA population used in this study). Nevertheless, while the differences in the global biomechanical parameters may be statistically insignificant between the three groups, the spatial distributions of wall stress are not identical. As shown in Figure 7, the wall stress distribution of the Predicted + NURBS model closely matches that of the Ground Truth model. This outcome appears to indicate that NURBS refinement of the outer wall segmentation effectively enhances the accuracy of the predictive model compared to an established ground truth observation, albeit at the cost of additional time devoted to NURBS refinement.

Recently, Chung *et al.*^40^ developed a framework to segment CTA images and estimate wall stress in AAAs. Their model was trained on 400 images from 10 AAAs to identify the lumen and outer wall boundaries, achieving a segmentation accuracy of 0.9980. In contrast, we used 147 AAAs from two different groups (symptomatic and asymptomatic), resulting in a segmentation accuracy of 0.9996. The output from Chung and colleagues’ model was used to generate a 3D surface mesh and extract morphological features for wall stress prediction using an Extra Trees regression model, with ground-truth wall stresses obtained from FEA. Their method achieved a coefficient of determination of 0.980 resulting from the comparison of the predicted wall stress with the ground truth. A comparison of our Predicted + NURBS SAWS with the Ground Truth SAWS resulted in a coefficient of determination of 0.9977. In contrast to the approach used by Chung and co-workers, we did not use a machine learning algorithm to estimate the wall stress. Instead, we directly solved the force balance equations for the predicted geometry (using NEMA), introducing less uncertainty in the final predictions and overcoming the FEA limitation of requiring patient-specific material properties. The automatic segmentation method described in^40^ requires 15 seconds per AAA, compared to our model’s 1.19 seconds per AAA (17 milliseconds per image) while using GPUs of similar performance (RTX 2080Ti vs. RTX A4500).

BioPARR is an open source software for the estimation of AAA wall stress and rupture potential index^41,42^. It uses a procedure similar to that of the present work for the estimation of wall stress, in that it overcomes the need for predefined material properties by applying a very stiff material^29^). However, it relies on multiple tools to build a complete pipeline. For example, segmentation is performed semiautomatically and takes approximately 45 minutes per case, which is substantially longer than our proposed segmentation model. Furthermore, an Abaqus license^43^ is required to compute the wall stress. In contrast, NEMA is executed with a MATLAB script in the final step of our modeling pipeline. In the present work, we describe a completely automatic pipeline that can estimate wall stress without statistically significant differences in wall stresses calculated directly from the predicted segmentations of CTA images. Furthermore, wall stress is estimated using the nonlinear elastic membrane analysis method, which is free from having to specify predefined material properties, as expected in traditional FEA-based methods.

The present work is subject to several important limitations, one of which is the reliance on contrast-enhanced CT images. To address this, future work will aim to incorporate unenhanced images into the training dataset as they become available. This is especially critical for AAA patients who may experience mild to severe adverse reactions to contrast media^44^ or exhibit renal insufficiency. Furthermore, increasing the training data set to incorporate a more extensive range of clinical presentations in CT exams would help the model perform better in more complex AAA cases. Future efforts will also focus on recognizing the inner wall boundary in abdominal CTA images, a problematic task existing models can only handle with user intervention. Another limitation is the use of a NEMA-based approach for biomechanical analysis. Although traditional stress analysis methods have the inherent limitation of unknown patient-specific material properties for the wall and ILT, a NEMA-based estimation of wall stress ignores bending stresses and stress gradients across the wall, which are considered by traditional forward FEA approaches.

## Conclusion

This study addresses the need for automatic and reliable AAA segmentation in contrast-enhanced CTA images while generating in-silico AAA models for shape modeling and wall stress estimation without user intervention. Using a patch-based dilated U-Net architecture, our methodology successfully overcomes key limitations of earlier CNN designs. The model accurately identifies the boundary of the AAA outer wall (with an IOU score of 0.9411 and a DSC of 0.9695) and achieves precise segmentation in 17 ± 0.02 milliseconds per image. This combination of precision and speed is especially valuable for clinical decision-making in AAA management. To further enhance the model’s effectiveness, we incorporated a NURBS-based tool, which enables precise modification of the outer wall boundary and allows for simple user interaction if desired. The combination of an advanced deep learning algorithm with NURBS produces a robust solution for the use of in-silico AAA models for wall stress estimation using NEMA, which has a high translational potential as part of a rupture risk assessment modeling pipeline. The framework can be easily adapted for the segmentation and biomechanical analysis of other vascular systems, extending its utility beyond AAA biomechanical analysis.

## Data Availability

The data set used in this study is not publicly accessible due to the regulations imposed by IRBs in the respective clinical centers. However, it can be obtained by making a reasonable request to coauthor E.F.
